# The Indigenous Australian Malnutrition Project: the burden and impact of malnutrition in Aboriginal Australian and Torres Strait Islander hospital inpatients, and validation of a malnutrition screening tool for use in hospitals—study rationale and protocol

**DOI:** 10.1186/s40064-016-2943-5

**Published:** 2016-08-08

**Authors:** Natasha F. Morris, Simon Stewart, Malcolm D. Riley, Graeme P. Maguire

**Affiliations:** 1 Department of Epidemiology and Preventive Medicine, Monash University, Melbourne, VIC Australia; 2 Baker IDI Heart and Diabetes Institute, PO Box 1294, Alice Springs, NT 0871 Australia; 3 NHMRC Centre Research Excellence to Reduce Inequality in Heart Disease, Mary MacKillop Institute for Health Research, Australian Catholic University, Melbourne, Australia; 4 CSIRO Food, Nutrition and Bio-Based Products, PO Box 10041, Adelaide BC, SA 5000 Australia; 5 James Cook University, School of Medicine and Dentistry, Townsville, QLD Australia; 6 Baker IDI Heart and Diabetes Institute, 75 Commercial Road, Melbourne, VIC 3084 Australia

**Keywords:** Indigenous Australians, Malnutrition, Malnutrition screening, Australian Nutrition Tool, Malnutrition screening tool, Subjective Global Assessment

## Abstract

**Background:**

Malnutrition is associated with adverse outcomes for hospital inpatients and is a significant economic burden on hospitals. Malnutrition is frequently under-recognised in this setting and valid screening and early diagnosis are important for timely nutritional management. Aboriginal Australian and/or Torres Strait Islander peoples (Indigenous Australians) are likely to be at increased risk of malnutrition due to their disproportionate burden, pattern and age-distribution of chronic diseases. Despite this increased risk, the burden and impact of malnutrition in Indigenous Australians is poorly understood. Furthermore, a suitable screening tool has not been validated for this vulnerable patient group. The aim of this study is to determine the burden of malnutrition, understand its impact, and validate a malnutrition screening tool for Indigenous Australian inpatients.

**Methods:**

This project involves cross-sectional, prospective cohort and diagnostic validation methodologies to assess the burden and impact of malnutrition and to validate a malnutrition screening tool. A target of 752 adult Indigenous and non-Indigenous Australian inpatients will be recruited across three different public hospitals in the Northern Territory and far north Queensland of Australia. Cross-sectional data collection will be used to determine the prevalence of malnutrition using the Subjective Global Assessment and to stratify participants based on the International Consensus Guideline Committee malnutrition aetiology-diagnostic framework. Subjects will then be followed prospectively to measure short and long-term health outcomes such as length of hospital stay, in-hospital mortality, 30-day and 6-month readmission rates. Finally, the utility of a new screening tool, the Australian Nutrition Tool, will be assessed against an existing screening tool, the malnutrition screening tool, used in these settings and the malnutrition reference standard, the Subjective Global Assessment.

**Discussion:**

Indigenous Australians continue to experience poorer levels of health than non-Indigenous Australians and issues such as food insecurity, poor diet, and a disproportionate burden of chronic disease play a key contributing role for malnutrition in Indigenous Australians. To improve the health and hospital outcomes of Indigenous and non-Indigenous Australians, it is important that patients are routinely screened using a validated screening tool. It is also imperative that the burden and impact of malnutrition is properly understood, and fully appreciated, so that early and appropriate nutritional management can be provided to this group of hospital patients.

## Background

### Defining malnutrition

Malnutrition is defined by the American Society of Parenteral and Enteral Nutrition (A.S.P.E.N.) as any “any nutritional imbalance” (White et al. 2012, p. 730). More specifically, malnutrition can refer to a state of under-nutrition (from insufficient intake or impaired utilisation), or over-nutrition (from excessive calorie intake and/or inadequate physical activity) (Escott-Stump 2012). Either state leads to altered body composition and function (Saunder et al. 2010). It is important however, that the term ‘under-nutrition’ is not confused with being underweight (e.g. often represented by Body Mass Index [BMI] <18.5 kg/m^2^). Under-nutrition is a term used synonymously with protein-energy deficiency, where there is a loss of body cell mass with signs of subcutaneous fat loss and skeletal muscle wasting and therefore (Escott-Stump 2012; Saunder et al. 2010); under-nutrition can occur irrespective of individuals’ BMI (Saunder et al. 2010). Malnutrition, in this study protocol, refers to ‘under-nutrition’, meaning protein-energy malnutrition (PEM) (Escott-Stump 2012).

### Causes of malnutrition

PEM is a condition caused by a number of inter-related factors. These factors include issues such as food insecurity due to poor social determinants of health; high-energy but nutrient diets; risk factors such as alcohol and illicit substance abuse; acute or chronic disease including mental health disorders; or acute injury such as burns and trauma (White et al. 2012; Escott-Stump 2012; Saunder et al. 2010; Jensen et al. 2009). Individuals at highest risk for malnutrition include the elderly, especially those with dementia; individuals suffering from chronic disease/s where they are not meeting their protein-energy requirements (Escott-Stump 2012); or individuals from vulnerable population groups such as Aboriginal Australian and/or Torres Strait Islander peoples (Indigenous Australians) (Browne et al. 2014; Clifford et al. 2010).

### Burden and impact of protein-energy malnutrition

**Fig. 1:**
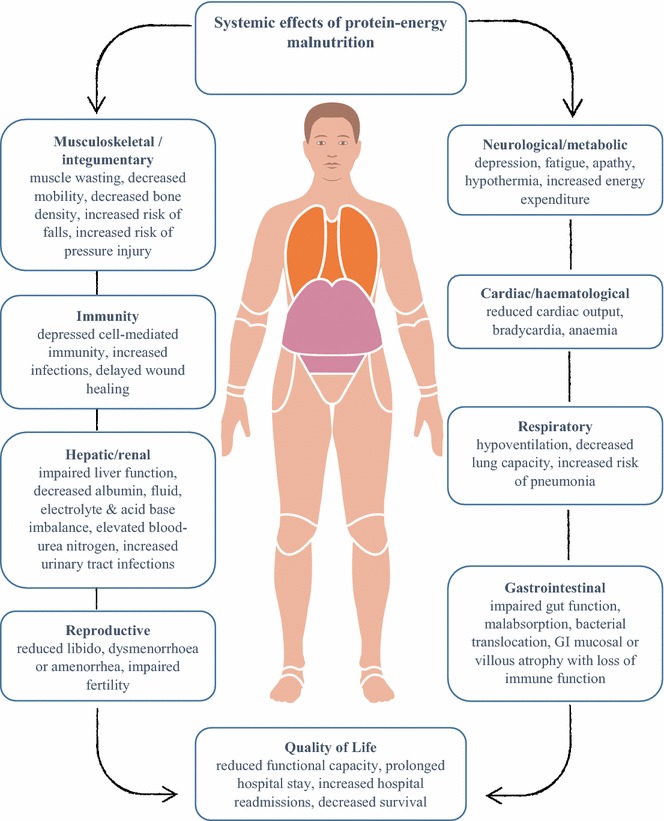
The systemic effects of malnutrition

Rates of hospital inpatient PEM vary greatly between surveys depending on the patient population and the methods used to diagnose malnutrition. In recent hospital-based surveys in Canada and Australia, using the Subjective Global Assessment (SGA), the prevalence of PEM in adult inpatients has been reported as ranging from 17 to 58 % (Allard et al. 2015; Ultang et al. 2012; Gibson et al. 2012). Compared to well-nourished patients, surveys also demonstrated that malnourished patients had an increased length of hospital stay, higher rates of in-hospital mortality, more frequent hospital readmissions, and overall poorer longer-term survival rates (Allard et al. 2015; Agarwal et al. 2013; Lim et al. 2012; Norman et al. 2008; National Collaborating Centre for Acute Care [NICE] 2006). These adverse outcomes are likely to be related to the systemic effects of malnutrition (see Fig. 1). Some of the main adverse and costly events associated with PEM include: increased risk of hospital-acquired infections; delayed wound healing; increased risk of falls; and increased risk of pressure injuries (Escott-Stump 2012; Barker et al. 2010; Banks et al. 2010; Saunder et al. 2010, Norman et al. 2008).

The financial impact of malnutrition is frequently underappreciated and often underestimated. Due to the lack of recognition, diagnosis and poor documentation, it is likely that malnourished patients are often not identified and coded correctly against ICD-10-AM criteria, which can result in a loss of hospital activity-based funding (Agarwal et al. 2013; Rowell and Jackson 2011; Banks et al. 2010). As a follow-up from the Australasian Nutrition Care Day Survey, malnourished patients’ medical records were audited (Agarwal et al. 2012). The results showed that only 16 % of malnourished patients had a separation code for malnutrition noted. Furthermore, out of 52 hospitals participating in the survey, 40 % of the hospitals failed to identify and code any malnourished patients (Agarwal et al. 2012).

The loss of activity-based funding associated with under-recognition and reporting of malnutrition was demonstrated in a public hospital in Victoria, Australia where malnutrition was estimated to add an additional annual cost of AUD $14.3 million (US $10.3 million) at 2015 valuations (Rowell and Jackson 2011). However, this cost was likely to represent a significant underestimate with only 1.9 % of inpatients being coded as malnourished compared to nearly one-third of adult inpatients being assessed as malnourished in more recent Australian surveys (Agarwal et al. 2012).

### Malnutrition screening

Systems to detect PEM or malnutrition risk in hospital inpatients has been shown to be variably implemented. Screening for malnutrition typically focuses on unintentional weight loss and sub-optimal oral intake in the last 6 months (Elia 2015). Due to a significant proportion of inpatients being at-risk of malnutrition, identifying at-risk or malnourished patients early in the admission process using a validated Malnutrition Screening Tool is best practice (Elia 2015; Dietitians Association of Australia [DAA] 2009; NICE 2006). It is advocated that early detection will facilitate early and comprehensive nutritional assessment and management by trained health providers, such as a dietitian (Elia 2015; Rowell and Jackson 2011; NICE 2006). The Malnutrition Screening Tool (MST) (see Table 1), developed in Australia, is a widely used malnutrition screening tool validated for the general adult inpatient population (van Bokhorst-de van der Schueren et al. 2014; Ferguson et al. 1999). Although the MST has demonstrated high sensitivity and specificity in different hospital inpatient populations (i.e. medical, surgical, and oncology inpatients) (van Bokhorst-de van der Schueren et al. 2014), the MST has not been validated for the Indigenous Australian population. The MST poses potential cultural and linguistic barriers that may potentially influence the reliability and validity of the tool (Shaw et al. 2015; Gibson et al. 2012; Clifford et al. 2010; Frew et al. 2010). These include patients needing to understand, and being able to recall, questions relating to changes in appetite and/or weight loss in the last 6 months (Fang et al. 2013; Ferguson et al. 1999). Furthermore, depending on local hospital protocols, a patient who is ‘unsure’ about recent unintentional weight loss may still prompt an, often unnecessary, referral to dietetic services.

**Table 1:** Malnutrition screening tool adapted from Ferguson et al. 1999

Malnutrition screening tool (MST)		
*Question 1* (*a*)		
Have you lost weight recently?	No (go to question 2)	Score 0
	Unsure (go to question 2)	Score 2
	Yes (go to question 1b)	Score –
*Question 1* (*b*)	0.5–5.0 kg	Score 1
If yes, how much weight (kg) have you lost?	5.1–10.0 kg (or unsure)	Score 2
	10.1–15.0 kg	Score 3
	15.1 or more	Score 4
	No	Score 0
*Question 2*	Yes	Score 1
Have you been eating poorly because of a decreased appetite?		
*MST score* (*Q1* + *Q2* + *Q3*)		

Due to these potential cultural and linguistic barriers, and the need for a reliable malnutrition screening tool for Indigenous Australians, the Australian Nutrition Tool (ANT, shown in Table 2) has been designed specifically for this project (Morris et al. 2015). The main differences between the MST and ANT are that patients are questioned about food intake rather than decreased appetite; weight loss is measured as a categorical variable rather than numerical; and ANT contains a third criterion where the health provider makes an assessment whether the patient looks undernourished (i.e. assessing for signs of subcutaneous fat loss, muscle wasting and/or poor skin integrity).

**Table 2:** The Australian Nutrition Tool (ANT) (Morris et al. 2015)

The Australian Nutrition Tool© (ANT)		
*Question 1*		
Do you think you have been eating enough food (or tucker) lately?	Yes	Score 0
	A little bit *or* not sure	Score 1
	No	Score 2
*Question 2*		
Do you think you have lost weight recently without trying?	No	Score 0
	Not sure	Score 1
	Yes	
If yes, how much weight do you think you have lost?	A little bit	Score 1
	A lot	Score 3
*Health provider assessment*		
Does the patient look undernourished?	No	Score 0
	A bit or not sure	Score 1
	Yes	Score 2
*ANT score* (*Q1* + *Q2* + *Q3*)		
*Nutrition Screening Scale*	*Action*	
Score 0–1	Weigh patient and rescreen in 7 days	
Score 2	Weigh patient, refer to hospital malnutrition action plan or policy, rescreen in 7 days	
Score ≥ 3	Weigh patient and refer to a dietitian	

### Diagnosing malnutrition

The diagnosis of malnutrition in hospital inpatients is further limited by the lack of an accepted gold-standard diagnostic definition (White et al. 2012; Jensen et al. 2009; Jensen and Wheeler 2012). According to the International Classification of Diseases-10-AM (ICD-10-AM), adult malnutrition is classified according to a BMI of ≤18.5 kg/m^2^; or a certain percentage of weight loss, suboptimal oral intake, and evidence of subcutaneous fat loss and/or muscle wasting (Australian Consortium for Classification Development 2015). It has been argued that the diagnosis of PEM is more complex than these ICD-10-AM criteria and a more global and systemic assessment of at-risk patients is required (White et al. 2012; Jensen et al. 2009, 2010).

A more comprehensive system for malnutrition assessment and diagnosis would incorporate the patients’ clinical diagnosis; past medical history (including communicable and non-communicable diseases); clinical assessment of weight history (i.e. loss of weight); changes to food intake (i.e. a sub-optimal intake); gastrointestinal signs and symptoms (i.e. nausea, vomiting, diarrhoea and/or anorexia); functional capacity (i.e. ability to complete day-to-day activities); a physical assessment for signs of subcutaneous fat loss and skeletal muscle wasting; anthropometric measures such as BMI; hand-grip strength; and blood chemistry data (Malone and Hamilton 2013; Jensen et al. 2010; Lochs et al. 2006). One such assessment tool is the SGA (Detsky et al. 1987). The SGA remains a superior nutritional assessment tool for detecting malnutrition in acute care settings (da Silva Fink et al. 2015; Steenson et al. 2013). A recent systematic review concluded that this tool had higher reliability and superiority than other nutrition assessment tools to detect malnutrition (da Silva Fink et al. 2015). Furthermore, and more recently, central to the diagnosis of malnutrition and evaluation of blood chemistry data, are inflammatory markers (such as C-Reactive Protein) due to the utilisation of proteins from the skeletal muscle in malnourished states (Jensen et al. 2010; Malone and Hamilton 2013; Lochs et al. 2006). Elevated inflammatory markers in malnutrition relate, in part, to the process of skeletal muscle proteins being utilised to meet metabolic requirements with resulting cytokine-mediated inflammation (White et al. 2012; Jensen et al. 2009, 2010; Malone and Hamilton 2013; Lochs et al. 2006).

### Nutritional health determinants of Indigenous Australians

Indigenous Australians experience well documented poorer health and lower life expectancy compared with non-Indigenous Australians (Australian Institute of Health and Welfare [AIHW] 2015; Browne et al. 2014; Clifford et al. 2010). Indigenous Australians experience a disproportionate burden of nutrition-related disorders and risk-factors (Browne et al. 2014; Clifford et al. 2010). For example, in 2013–2014, Indigenous Australian adults were 3.7 times more likely to have chronic renal disease; 3.3 times more likely to have diabetes; and 1.2 times more likely to have cardiovascular disease than non-Indigenous Australians (AIHW 2015). These conditions in turn, increase patients’ risk for malnutrition, which adversely affect clinical outcomes (AIHW 2015; Norman et al. 2008). Furthermore, Indigenous Australians also experience disproportionate nutrition-related risk-factors for chronic disease(s), and are more likely to be overweight (BMI ≥ 25.0 kg/m^2^) or obese (BMI ≥ 30.0 kg/m^2^) compared to non-Indigenous Australians (Australian Bureau of Statistics [ABS] 2012–2013).

Poor nutrition (i.e. poor fruit and vegetable intake and high consumption of high-energy but nutrient poor foods) is a factor in the prevention and management of chronic disease for Indigenous Australians (Pettrigrew et al. 2015; Browne et al. 2014; Clifford et al. 2010). Given the disproportionate burden of nutrition-related diseases in the Indigenous Australians population, early and reliable malnutrition screening leading to earlier diagnosis, referral and management is an important and often ignored strategy for improved health outcomes. Although the burden and impact of malnutrition in a range of adult hospital inpatient populations has been widely reported elsewhere (Allard et al. 2015; Corkins et al. 2014; Houng et al. 2014; Fang et al. 2013; Agarwal et al. 2012; Álvarex-Hermández et al. 2012; Lamb et al. 2009; Meijers et al. 2009; Westergren et al. 2009), the prevalence, burden and impact of malnutrition in adult Indigenous Australians has not been previously reported and is poorly understood. Therefore, the aims of this research project are to:

1. Determine the prevalence of malnutrition in adult Indigenous Australians and assess the different types of malnutrition Indigenous Australians may experience according to a malnutrition aetiology-diagnostic framework;
2. Assess the health and health care impact of malnutrition in adult Indigenous Australians compared to non-Indigenous Australians by assessing length of hospital stay, in-hospital survival, 30 day and 6 month readmission and survival; and
3. Assess the validity of a culturally appropriate screening tool for Indigenous Australians as well as other patients with linguistic barriers where English may not be a first language.

## Methods

**Fig. 2:**
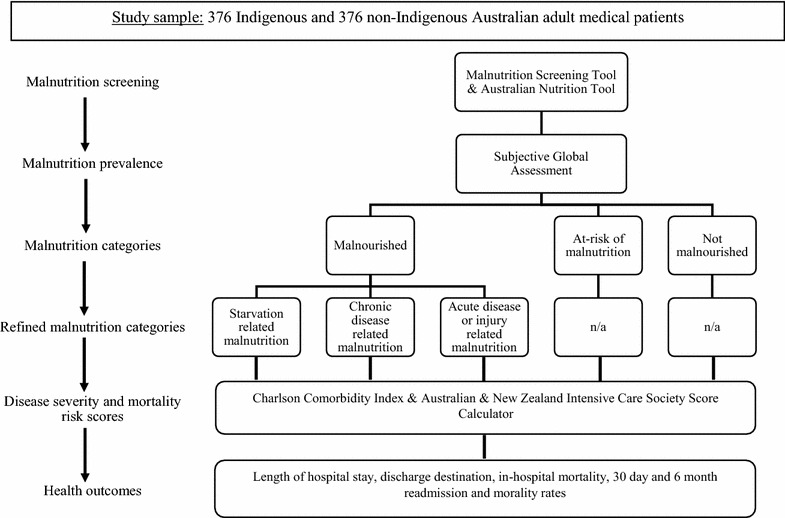
Indigenous Australian Malnutrition project study design

The Indigenous Australian Malnutrition study incorporates cross-sectional, prospective cohort and diagnostic validation studies to assess the prevalence and impact of PEM and to validate a malnutrition screening tool (see Fig. 2). The project will be conducted in two public hospitals (Alice Springs Hospital and the Royal Darwin Hospital) in the Northern Territory and one public hospital (Cairns Base Hospital) in the far north of Queensland, Australia. The project sampling frame will include all non-elective community and residential-care based admissions to adult internal medicine units (including medical sub-specialties). Indigenous (people identified by patient registration data or who self-identify as Aboriginal Australian and/or Torres Strait Islander) and non-Indigenous Australians aged 18 years and over who are able to provide informed consent (or who have a carer who is able to consent) will be invited to participate. Patients excluded from the study will include those under the age of 18 years, medical patients admitted directly to an intensive care unit, inter-hospital transfers, patients (or where appropriate, carers) unwilling or unable to provide informed consent, pregnant or lactating women, and non-medical admissions (i.e. surgical, orthopaedic, paediatric, routine renal dialysis and mental health admissions).

### Cross-sectional study of the prevalence of inpatient malnutrition

All consenting participants will be screened for malnutrition risk (by NM and an independent dietitian) using the MST and ANT. The ANT was developed with the involvement of dietitian experts from each of the three study sites, Indigenous and non-Indigenous health providers (including Aboriginal Liaison Officers), and academic experts.

**Fig. 3:**
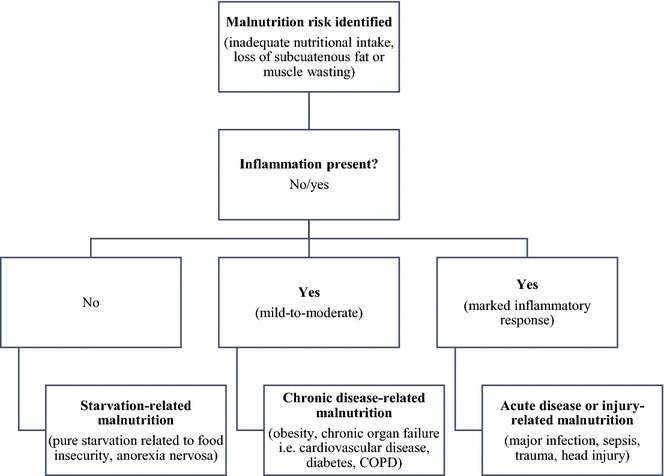
ICGC malnutrition aetiology-diagnostic framework

Following screening, all participants, irrespective of MST and ANT scoring, will have a diagnostic assessment for malnutrition using the SGA (Jensen et al. 2009). The prevalence of PEM will be determined using the SGA and stratified using existing SGA criteria: well-nourished (SGA A); mild-to-moderate malnutrition (SGA B); and severe malnutrition (SGA C). Participants who are assessed as well-nourished (SGA A) will be assessed for malnutrition risk based on SGA risk-factors including (i.e. unintentional weight loss within the last 6 months and suboptimal food intake). All participants will undergo further nutritional assessment using the World Health Organisation (WHO) BMI classification (WHO 2016); WHO waist-hip ratio (WHO 2008); mid-upper arm circumference, and hand-grip strength measurement using standard measurement techniques (Stewart 2015). Malnourished patients will be stratified into malnutrition categories based on the A.S.P.E.N. and European Parenteral and Enteral Nutrition (ESPEN) International Consensus Guideline Committee (ICGC) malnutrition aetiology-diagnostic framework (see Fig. 3) (White et al. 2012; Jensen et al. 2010). The ICGC classifies malnutrition into three main types: starvation-related malnutrition; chronic-disease related malnutrition; and acute disease or injury-related malnutrition (White et al. 2012; Jensen and Wheeler 2012; Jensen et al. 2009, 2010).

### Prospective cohort study to assess the impact of malnutrition

The second component of this study will be to assess the impact of PEM on hospital inpatients and the hospital health care system. This will be assessed by evaluating the influence of PEM (including; stratification by severity and aetiology) on length of hospital stay, in-hospital mortality, discharge destination, and 30 days and 6 months readmission and survival using hospital and jurisdictional death registry data.

Comorbidities, the severity of the presenting illness and other contributors to health outcome will be controlled for using the Charlson Comorbidity Index (Charlson et al. 1987), the Australian New Zealand Intensive Care Society (ANZICS) score calculator and multivariate techniques. The Charlson Comorbidity Index particularly focuses on patient age and existing comorbidities that have been previously shown to influence survival (Charlson et al. 1994). The ANZICS score calculator focuses on acute illness severity and include the Acute Physiology and Chronic Health Evaluation (APACHE) II score; APACHE II risk of death; Simplified Acute Physiology Score (SAPS) II score; and SAPS II risk of death (ANZICS Research Centre 2010).

### Diagnostic validation study of screening tools

The final component of this study will be an assessment of the validity of the screening tools in this setting to detect PEM in inpatients. Results for the existing MST and new ANT will be compared to the SGA. Standard diagnostic validation techniques will be utilised (see statistical analysis below) to identify suitable scoring cut-offs and to determine overall utility as a screening tool in this setting.

### Sample size analysis

The sample size required to detect a 10 % absolute difference in the prevalence of malnutrition in Indigenous as compared with non-Indigenous Australians (based on 2 equal sized groups, an assumed prevalence of malnutrition of 30 % in Australian adult inpatients (Agarwal et al. 2013), power of 80 % and two-sided alpha of 0.05) is 752 (i.e. 376 in each group).

### Statistical analysis

Data will be analysed using Stata^®^ 14 (StataCorp College Station, TX, USA). Descriptive analysis of participants will utilise standard univariate techniques and will be reported as percentages with 95 % confidence intervals (95 % CI), means with standard deviations (SD) or medians with interquartile range depending on the data format and distribution. Comparisons between Indigenous and non-Indigenous participants will be undertaken using χ^2^ for categorical data and Student’s *t* Test or Mann–Whitney U test for continuous normally distributed data or non-normally distributed data respectively. A p value <0.05 will be taken to indicate statistical significance and all tests will be two-sided. The utility of the MST and ANT to detect PEM as measured by the SGA will be investigated using diagnostic test analysis including determining sensitivity and specificity, and positive and negative predictive values. Optimal scoring cut-offs for the MST and ANT will be determined using receiver operator characteristic (ROC) analysis.

The impact of PEM on the outcomes listed above will be first assessed using bivariate analysis for length of hospital stay, in-hospital mortality, discharge destination, 30 days and 3 months readmission. Survival to 6 months post-discharge will be presented using Kaplan–Meir curves and analysed using the log rank test to compare survival in patients with and without PEM, and in Indigenous and non-Indigenous Australians.

Multivariable linear, logistic and Cox proportional hazard models will be developed to identify independent factors associated with outcome measures. These will use a backwards stepwise approach including in the first model all factors associated with a particular outcome variable using bivariate analysis with a p value <0.1. Factors with a p value ≥0.05 will be progressively removed from the models starting with those variables with a regression co-efficient closest to 0 or an odds or hazard ratio closest to 1. Final models will be limited to predictive factors with significant coefficients (p < 0.05).

Diagnostic validation analysis will first involve the assessment of concordance between the MST and ANT by the technique of Lin (1989) that assesses the agreement between two different continuous measures. Mean difference (bias) and limits of agreement will be determined using the techniques of Bland and Altman (1999). Cut-offs for scores for both screening tools for excluding and diagnosing malnutrition will be determined from receiver operating characteristics (ROC) curve analysis with multivariate regression analysis used to determine whether patient factors affect the relationship between the screening tool score and SGA assessment.

### Ethics approval

This project has been approved by the Central Australian, Menzies, Far North Queensland, and Monash University Human Research Ethics Committees.

## Discussion

The study outlined in this paper aims to provide a new and detailed perspective on the burden, impact and diagnosis of PEM in a high income country setting and in an underserved population, in this instance, Indigenous Australians. The prevalence of PEM and those at-risk of malnutrition is significant in hospital inpatients (Allard et al. 2015; Agarwal et al. 2012, 2013; Ultang et al. 2012; Gibson et al. 2012). PEM, in turn, has been associated with a number of adverse clinical outcomes (Allard et al. 2015; Agarwal et al. 2012, 2013; Escott-Stump 2012; Lim et al. 2012; Rowell and Jackson 2011; Saunder et al. 2010). Whilst the cost of PEM is substantial for both patients and the health care system (Agarwal et al. 2013; Lim et al. 2012; Barker et al. 2010; Rowell and Jackson 2011; Norman et al. 2008), understanding of its burden and impact remains limited, particularly in underserved populations such as Indigenous Australians. We hypothesise that the burden and impact of malnutrition in Indigenous Australians is likely to be greater and more severe than for non-Indigenous Australian inpatients. We also anticipate that the type of PEM that Indigenous Australian inpatients present with is more likely to be chronic disease-related. Such stratification of malnutrition into different aetiology types is important to allow a tailored response to management. Whilst documenting the burden and impact of malnutrition is important this information must also, where appropriate, be utilised to enhance health care practice. While the Dietitians Association of Australia recommend that all hospital patients are screened within 24 h of hospital admission (DAA 2009), this is variably implemented (Green and James 2013). In addition, whilst such screening must necessarily use a reliable malnutrition screening tool that has been validated for a specific patient population this is not possible for Indigenous Australians where no such validated tool currently exists. By validating a new malnutrition screening tool (ANT), we hope to enhance and inform the detection and management of PEM in Australia in general and for Indigenous Australians. In summary, the Indigenous Australian Malnutrition project will provide new insights into PEM in adult Indigenous and non-Indigenous Australians in the acute care setting and seek to improve and simplify diagnosis with the validation of a new malnutrition screening tool (ANT) for the detection of PEM in Indigenous Australians.

## Abbreviations

ANT: Australian Nutrition Tool
APACHE: Acute Physiology and Chronic Health Evaluation
ANZICS: Australian New Zealand Intensive Care Society
A.S.P.E.N.: American Society of Parenteral and Enteral Nutrition
BMI: Body Mass Index
CCI: Charlson Comorbidity Index
ESPEN: European Society of Parenteral and Enteral Nutrition
HREC: Human Research Ethics Committee
ICD: International Classification Of Diseases
ICGC: International Consensus Guideline Committee
LoS: Length of stay
MST: malnutrition screening tool
PEM: protein energy malnutrition
SAPS: Simplified Acute Physiology Score
SGA: Subjective Global Assessment
